# Increases in One-year Mortality Risk Among Chronic Skin Ulcer Patients During the Period 1980–2020

**DOI:** 10.2340/actadv.v106.43626

**Published:** 2026-02-10

**Authors:** Jenni E. SALENIUS, Teea T. SALMI, Minna SUNTILA, Heini HUHTALA, Teija KIMPIMÄKI

**Affiliations:** 1 Faculty of Medicine and Health Technology, Tampere University, Tampere; 2 Department of Dermatology, Tampere University Hospital, Wellbeing Services County of Pirkanmaa Tampere; 3 Faculty of Social Sciences, Tampere University, Tampere, Finland

**Keywords:** diabetes mellitus, leg ulcer, mortality, peripheral arterial disease, sepsis, skin ulcer

## Abstract

Chronic ulcer patients often suffer from multiple comorbidities and may even face increased risk of death. This study investigated short-term mortality of 5,230 ulcer patients treated in tertiary healthcare between 1980 and 2020, and their 15,594 matched references. Hazard risks (HR) for mortality were compared between patients with venous, arterial, mixed, vasculitic, and pyoderma gangrenosum ulcers (PG) and between 4 x 10-year study periods. One-year mortality risk was increased among all ulcer patients over the whole study period (HR 3.8), and it was highest among patients with vasculitic (HR 8.5), arterial (HR 7.0), and PG (HR 6.6) ulcers. During the 4 study decades the mortality HR of all ulcer patients increased from 2.3 to 4.9. However, among patients with vasculitic and PG ulcers, mortality risk decreased between the last decades observed. Among causes of death, highest 1-year HR for mortality from underlying causes of death was diabetes (HR 31.6), and of selected immediate causes of death, mortality from sepsis was notably increased (HR 22.1). In conclusion, patients with chronic ulcers were at almost fourfold increased risk of 1-year mortality and the risk increased over time. The study stresses the need for a multidisciplinary approach and effective treatment of underlying conditions.

## SIGNIFICANCE

This study investigated the short-term mortality risk of patients with chronic ulcers 1980–2020. Patients were found to have an almost fourfold increased risk of 1-year mortality, the risk being highest among those with vasculitic and arterial ulcers. Short-term mortality risk seemed to increase over time, except that among patients with pyoderma gangrenosum (PG) and vasculitic ulcers a recent decline in mortality was detected. Patients were notably at risk of death from diabetes, digestive, and ischaemic heart diseases. Therefore, a multidisciplinary approach and effective treatment of underlying long-term illnesses is crucial.

The risk of chronic ulcers increases with age (1) and consequently, along with population ageing, the number of patients with chronic ulcers has increased in recent decades (2). Further, there is a significant burden associated with chronic ulcers, as such patients frequently have multiple comorbidities, and, at worst, chronic ulcers may even increase mortality (3–8). We have previously established that the long-term mortality risk among chronic ulcer patients is increased regardless of age, gender, and ulcer aetiology (9). Obvious explanations for increased long-term mortality include high age and associated diseases, while ulcer complications such as infections and also complications related to ulcer treatment may expose patients to short-term mortality.

There are some data on the short-term mortality risk among chronic ulcer patients (6, 10–13). In these studies short-term mortality has been reported to be increased, particularly among patients with diabetic or pressure ulcers (5, 10, 13), and to some extent also among those with arterial ulcers (12). However, only a few studies have investigated short-term mortality among those with atypical ulcers, i.e., ulcer aetiologies other than vascular, diabetic, or pressure (7, 14–15). Moreover, while it is still unknown whether mortality of ulcer patients has changed over time, ulcer treatment has progressed as in recent decades new wound care products have been developed, and wound care techniques and treatment modalities have advanced (16). Also, holistic treatment has gained importance in wound care, and consequently multidisciplinary wound centres have been established and are recognized as the most efficient and cost-effective way to treat patients with chronic ulcers (17). On the other hand, as the population in general ages, the number of elderly patients with chronic ulcers increases, and comorbidities become more prevalent with advancing age. Over time, these factors may counteract the beneficial effects of improved care on mortality trends.

The present study is a continuum of our earlier study investigating long-term mortality among chronic ulcer patients (9). This study aims to explore the short-term mortality risk among chronic ulcer patients with different aetiologies. A further aim is to examine how the short-term mortality risk has evolved over time, particularly after the establishment of a multidisciplinary wound centre.

## MATERIALS AND METHODS

### Study population and protocol

The study comprised adult patients (≥ 18 years of age) with chronic ulcers diagnosed and treated as in- or outpatients at Tampere University Hospital, between 1 January 1980 and 31 December 2019, i.e., the study period. Inpatients were treated in the Department of Dermatology until 2012 and outpatients until 2016 (9), after which they were treated in the Wound Centre of Tampere University Hospital. The Wound Centre is a tertiary care unit providing multidisciplinary and comprehensive specialized care for patients with hard-to-heal chronic ulcers (18, 19).

The study patients were collected from Tampere University Hospital’s patient record system using the International Classification of Diseases versions 8–10 (ICD8–10) diagnostic codes for venous, arterial, mixed (i.e., venous ulcers with peripheral arterial disease), vasculitic, and pyoderma gangrenosum (PG) as well as unspecified chronic ulcers, as previously described (9, 20). The medical records of those patients with multiple, unspecified, or vasculitic ulcer diagnoses were reviewed manually to confirm the main diagnosis. If the correct ulcer diagnosis turned out to be diabetic foot, pressure, or acute ulcer, patients were excluded from the study, as were those without ulcers or having non-PG ulcers located elsewhere than in the lower extremities. In addition to the ulcer diagnoses, the data gathered from the patient record system included age, gender, and the index day, i.e., the time of the first ulcer diagnosis during the study period.

Ulcer patients were further divided into subcategories of patients with venous, arterial, mixed, vasculitic, or PG ulcers, and in these analyses only patients with ulcers with a single aetiology during the study period were included as previously described (9). Also, ulcer patients were divided into subgroups according to year of the index day, and in this analysis the following study decades were included: 1980–1989, 1990–1999, 2000–2009, and 2010–2019.

A reference group for ulcer patients was formed from the Finnish Digital and Population Data Services Agency, which is a central register for maintaining the residence details of Finland’s population. The reference group consisted of 3 subjects for each ulcer patient matched for sex, age, and place of residence on the index day. Dates of possible emigration for both ulcer patients and reference group were acquired from the Digital and Population Data Services Agency.

The follow-up time for each study participant began on the index day and ended on the date of death (see below), emigration from Finland, or the data collection date, 31 December 2020. The Regional Ethics Committee of Tampere University Hospital approved the study protocol (R21002R), and it conformed to the ethical guidelines of the Declaration of Helsinki.

### Mortality data

The personal identification codes of the ulcer patients and their reference group were used to acquire the information on the date and cause of death from Statistics Finland, which is Finland’s national statistical institute that maintains the registry of causes of death and covers more than 99% of all deceased residents of Finland since 1936 (21). Underlying and immediate causes of death were derived from the death certificate data. In the registry of Statistics Finland (21) the underlying causes of death are classified into 54 groups. In the analyses of the underlying causes of death in this study, the following groups with the largest number of ulcer patients were included: ischaemic heart disease, all malignancies, cerebrovascular diseases, dementia including Alzheimer’s disease, diabetes mellitus (all types), digestive diseases excluding all alcohol-related diseases and pneumonia. The registry codes the immediate causes of death according to ICD8-10. In the present study, sepsis and pneumonia were included as immediate causes of death within the analyses, because in the authors’ clinical experience and according to earlier research on chronic ulcers, these are commonly occurring complications among patients with chronic ulcers (4, 8, 13, 22).

### Statistical analysis

The data were analysed using SPSS Statistics 25.0 (IBM Corp, Armonk, NY, USA) and Stata 16.0 (StataCorp LLC, College Station, TX, USA). Median values and interquartile ranges (IQR) were used to describe continuous variables, and numbers and percentages to describe categorical variables. In all analyses, ulcer patients were compared with their respective reference groups using Cox proportional hazards regression models to evaluate hazard ratios (HR) and 95% confidence intervals (95% CI). When assessing different causes of death, the proportional hazards assumption was evaluated using both Kaplan–Meier curves and Schoenfeld residuals. Mortality from competing causes of death was considered using Fine & Gray’s proportional sub-hazards model, but was not included in the final results, because the values essentially remained unchanged.

## RESULTS

This study comprised altogether 5,230 ulcer patients and a reference group of 15,594 individuals. For 32 ulcer patients, not all 3 matched references were found. Among the ulcer patients and reference group 61.4% were females, and the median (IQR) age on the index day was 71.7 years in both groups (60.6–80.3). When only patients having ulcers with the same aetiology during the study period were included, the most common ulcer aetiology was venous insufficiency (61.3%) (**Table I**).

**Table I:** Demographic data on chronic ulcer patients with different aetiologies during the whole study period and in 10-year study periods

Ulcer diagnosis^a^	1980–2020 *n*=3,880	1980–1989 *n*=784	1990–1999 *n*=639	2000–2009 *n*=929	2010–2019 *n*=1,528
Venous ulcers, *n* (%)	2,379 (61.3)	753 (96.0)	416 (65.1)	559 (60.2)	651 (42.6)
Female, *n* (%)	1,591 (66.9)	540 (71.7)	299 (71.9)	373 (66.7)	379 (58.2)
Age^b^, years/median	72.2	69.3	70.7	74.8	74.5
Arterial ulcers, *n* (%)	801 (20.6)	11 (1.4)	144 (22.5)	194 (20.9)	452 (29.6)
Female, *n* (%)	360 (44.9)	8 (72.7)	67 (46.5)	101 (52.1)	184 (40.7)
Age^b^, y/median	76.7	72.5	76.0	78.3	76.4
Mixed ulcers, *n* (%)	174 (4.5)	6 (0.8)	23 (3.6)	57 (6.1)	88 (5.8)
Female, *n* (%)	109 (62.6)	3 (50.0)	17 (73.9)	39 (68.4)	50 (56.8)
Age^b^, years/median	80.5	71.4	76.5	83.0	80.2
Vasculitic ulcers, *n* (%)	354 (9.1)	13 (1.7)	44 (6.9)	71 (7.6)	226 (14.8)
Female, *n* (%)	219 (61.9)	7 (53.8)	25 (56.8)	42 (59.2)	145 (64.2)
Age^b^, years/median	70.2	56.7	66.2	65.0	74.4
Pyoderma gangrenosum ulcers, *n* (%)	172 (4.4)	1 (0.1)	12 (1.9)	48 (5.2)	111 (7.3)
Female, *n* (%)	101 (58.7)	1 (100)	7 (58.3)	28 (58.3)	65 (58.6)
Age^b^, years/median	57.9	55.3	57.6	60.3	57.1

a Including ulcer patients with ulcers of only 1 aetiology during the study period.

b Age on the index day.

**Fig. 1:**
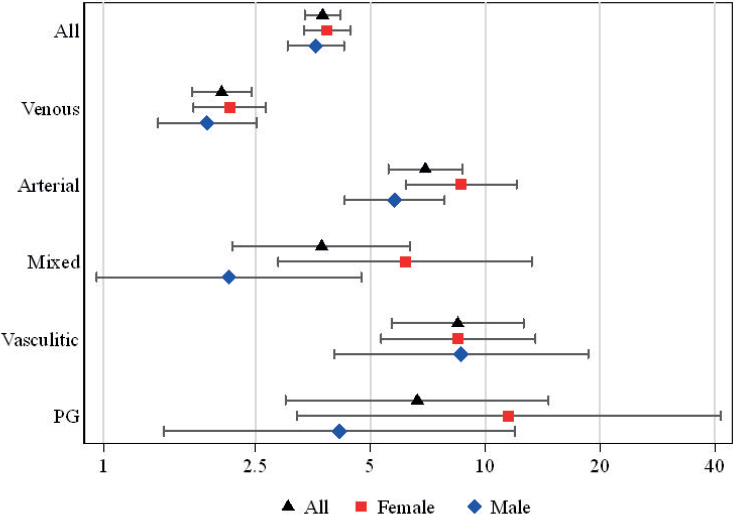
Hazard ratios and 95% confidence intervals for 1-year mortality in all ulcer patients and separately in females and males with different ulcer aetiologies during the period 1980–2020.

During the study period 71.8% of the ulcer patients compared with 54.1% of the individuals in the reference group died, and within 1 year after the index day 13.7% of the ulcer patients and 3.9 % of the reference group died. Among all ulcer patients, the 1-year HR for mortality for the whole study period was 3.8 (**Fig. 1** and Table SI). When mortality data were analysed separately for females and males, the 1-year HR for mortality was slightly higher for females (3.9) than males (3.6) when compared with their own references. When subgroups of ulcer patients with different ulcer aetiologies were studied, the highest 1-year HR for mortality was found among patients with vasculitic ulcers (8.5). When this subgroup analysis was performed separately for females and males, in females the highest 1-year HR for mortality was among patients with PG ulcers (11.5) and for males among patients with vasculitic ulcers (8.7).

**Fig. 2:**
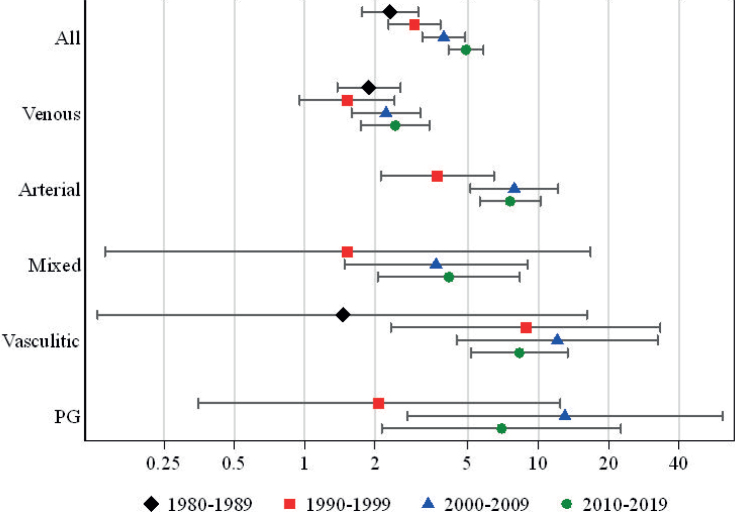
Hazard ratios and 95% confidence intervals for 1-year mortality in different ulcer aetiologies during the 4 study decades.

When the 4 study decades were investigated separately, the number of ulcer patients increased progressively from 984 between 1980 and 1989 to 1924 between 2010 and 2019. In the mortality analysis, the 1-year HR for mortality for ulcer patients gradually increased over time from 2.3 to 4.9 (**Fig. 2** and Table SII). When the 1-year mortality was assessed separately in female and male ulcer patients in the 4 study decades, HR for mortality was shown to increase in both genders: in females from 2.4 (1.7–3.4) to 5.5 (4.4–7.0) and in males from 2.1 (1.2–3.5) to 4.3 (3.3–5.5). Moreover, when mortality was investigated in the different study decades separately in 5 ulcer aetiology subgroups, HR for 1-year mortality increased in all ulcer aetiologies except that a marginal decrease was detected in arterial ulcers (from 7.9 to 7.6) and was more notable among PG (13.0–7.0) and vasculitic (12.1–8.3) ulcers in the last study decade.

In the analysis of the underlying causes of death, diabetes had the highest 1-year HR for mortality (31.6, 12.6–79.1) when all ulcer patients and the whole study period were included (**Table II**). Diabetes also had the highest HR for 1-year mortality among patients with arterial (72.2) and venous (12.4) ulcers, but among patients with mixed, vasculitic, and PG ulcers HR was highest for ischaemic heart diseases (6.3, 10.4, and 9.4 respectively).When selected immediate causes of death were analysed for all ulcer patients during the whole study period, the 1-year HR for sepsis mortality was 22.1 (5.6–74.0), and for pneumonia 4.8 (3.5–6.7). In further analysis including patients with different ulcer aetiologies, the highest 1-year HR for pneumonia mortality was among patients with vasculitic ulcers (37.8, 4.9–292.8). Due to the small numbers of cases, the 1-year HR for sepsis mortality could be evaluated only for patients with venous ulcers (9.2, 1.9–45.5).

**Table II:** Mortality rates, hazard ratios (HR), and 95% confidence intervals (CI) for 1-year mortality for the most common underlying causes of death in ulcer patients compared with their own reference groups

Diagnosis	All ulcers		Venous ulcers^a^ HR (95% CI)	Arterial ulcers^a^ HR (95% CI)	Mixed ulcers^a^ HR (95% CI)	Vasculitic ulcers^a^ HR (95% CI)	PG ulcers^a^ HR (95% CI)
	Mortality rate (10 000 py) (95% CI)	HR (95% CI)					
Diabetes^b^	Patients 8.7 (6.6–11.5) References 0.3 (0.1–0.7)	31.6 (12.6–79.1)*	12.3 (3.5–43.4)*	72.2 (9.7–536.7)*	NA	6.4 (0.6–70.9)	NA
Digestive diseases	Patients 5.2 (3.6–7.5) References 1.0 (0.6–1.6)	5.3 (3.0–9.5)*	2.3 (1.0–5.5)	15.6 (4.4–54.8)*	NA	9.7 (1.0–93.5)*	NA
Ischaemic heart diseases	Patients 33.1 (28.8–38.2) References 8.4 (7.2–9.9)	3.9 (3.2–4.8)*	2.2 (1.6–3.1)*	7.3 (4.8–11.0)*	6.3 (1.6–25.0)*	10.4 (4.4–24.6)*	9.4 (1.9–46.6)*
Pneumonia	Patients 2.1 (1.2–3.7) References 0.9 (0.5–1.4)	2.4 (1.1–5.0)*	1.8 (0.7–4.5)	6.9 (0.6–75.7)	NA	6.6 (0.6–73.2)	NA
Cerebrovascular diseases	Patients 8.0 (6.0–10.7) References 3.65 (2.9–4.6)	2.2 (1.5–3.2)*	1.7 (1.0–2.9)	3.8 (1.8–7.9)*	1.6 (0.3–8.8)	2.2 (0.4–13.1)	NA
Malignancies	Patients 16.8 (13.8–20.5) References 7.9 (6.7–9.3)	2.1 (1.7–2.8)*	1.1 (0.7–1.8)	3.6 (2.0–6.2)*	2.7 (0.8–9.0)	2.6 (1.1–5.9)*	2.1 (0.6–7.5)
Dementia	Patients 4.7 (3.2–6.8) References 2.9 (2.25–3.8)	1.6 (1.0–2.5)*	0.9 (0.4–1.9)	3.5 (1.4–8.7)*	NA	1.8 (0.3–9.8)	NA

a Including ulcer patients with ulcers of only 1 aetiology during the study period.

b Including all types of diabetes.

py: person-years; HR: hazard ratios; CI: confidence intervals; PG: pyoderma gangrenosum; NA: not applicable.

* *p* < 0.05.

## DISCUSSION

This study investigating the short-term mortality of 5,230 patients with chronic ulcers over 4 decades (1980-–2020) established that the patients with chronic ulcers faced an almost fourfold higher 1-year mortality risk than their reference group. Furthermore, the highest 1-year mortality risk was observed in patients with vasculitic, arterial, or PG ulcers. However, the mortality of vasculitic and PG ulcer patients was shown to decrease within the last decade after the establishment of a multidisciplinary wound centre.

Research conducted previously on the short-term mortality risk of chronic ulcer patients has mainly focused on patients with diabetic foot and pressure ulcers (10, 13), who were not included in this study investigating mortality in ulcer aetiologies with less available knowledge. Of the vascular ulcers, a few studies presented increased short-term mortality in patients with arterial ulcers (3, 11, 12), which is in alignment with our findings. Less is known about short-term mortality associated with venous ulcers, but in 1 study 16.1% of the patients with venous ulcers, compared with 2.9% of the total population, had died within 2 years (8), thereby corroborating the increased 1-year mortality among this patient group detected in our present study. Particularly scarce evidence is available on the short-term mortality of patients with atypical ulcers such as vasculitic and PG ulcers. However, 1 retrospective study with only 14 patients found markedly increased mortality in patients with rheumatoid arthritis and cutaneous vasculitic ulcers within the first 2 years of follow-up (conditional risk ratio 5.7) (14). The result is somewhat parallel with ours, demonstrating an almost ninefold increased HR for 1-year mortality among patients with vasculitic ulcers. Further, 2 studies present the Kaplan–Meier survival curves of PG patients compared with those of controls, which diverge directly after PG diagnosis (15, 23). Overall mortality of PG patients in these studies was increased two- to threefold, which is less than in our study (HR 6.6) focusing on short-term mortality. Consistently and rather interestingly, in our earlier study, with a partly overlapping cohort, long-term mortality of all ulcer patients was likewise significantly lower than the 1-year mortality detected in this study (HR 1.7 vs. 3.8) (9). In our earlier study we did not investigate vasculitic and PG patients separately, but long-term HR for mortality in patients with atypical ulcers was 2.2, and lower risks than in this study were also detected in groups consisting of patients with arterial, mixed, and venous ulcers. This implies that the risk of death is at its peak immediately after patients are diagnosed with ulcers, and this should be taken into consideration in clinical practice. Further, short-term mortality risk seems to be particularly increased in patients with vasculitic ulcers as found in this study, while long-term mortality is more increased in those with arterial ulcers (9).

In the present study, female patients with chronic ulcers exhibited a slightly greater 1-year mortality risk than males when compared with their own reference groups. Conversely, in another study on all-cause mortality risk among chronic ulcer patients, male patients had a higher mortality risk than females, and, rather intriguingly, a similar finding was observed in our earlier long-term mortality study with partly overlapping study cohort (8, 9). One explanation for the differences between our present study and others is that this study did not include diabetic foot ulcers, which are known to be associated with increased mortality and are more common in males (13, 24). However, this does not explain the discrepancy between our 2 studies, and explanations for this difference remain obscure. Nonetheless, in this study the highest mortality risk was associated with atypical ulcers in both males and females, but in males the highest HR for mortality (HR 8.7) was in those with vasculitic ulcers whereas in females it was in those with PG ulcers (HR 11.5).

This study reported an almost twofold increase in the number of patients, which could imply that the number of patients with hard-to-heal ulcers has increased over time. The advances occurring during our study period include improved knowledge and education in wound care (16) and evolvement of treatment modalities, particularly concerning vascular ulcers (25, 26). As an important achievement in our hospital, a multidisciplinary wound centre was established during the study period (18, 19). Despite these advances, the HR for short-term mortality was shown to double between our first and last study decades. One explanation for the increasing mortality could be the increasing proportion of patients with arterial ulcers, which had the second highest mortality of the ulcer aetiologies in our study population. However, similar increasing risks were detected when mortality trends were investigated separately in different ulcer aetiologies, with the exception of a notable decline in short-term mortality in patients with vasculitic and PG ulcers between the last 2 study decades. Severe cases of vasculitis and PG often benefit from multidisciplinary treatment by a dermatologist, internist, plastic surgeon, and possibly also a vascular surgeon and a gastroenterologist. Also, due to immunosuppressive treatments used in these conditions, these patients are at risk of infections. In this study patients with vasculitic ulcers had significantly higher risk of pneumonia mortality than ulcer patients in general (HR 37.8 vs 4.8), emphasizing that a specialist in infectious diseases is a vital member of the treatment team. Thus, the establishment of a multidisciplinary wound centre may have contributed this decline in mortality among these patient groups. However, a multidisciplinary approach is also of value for other patients with chronic ulcers, as recognized by the European Wound Management Association (EWMA) (27). As evidence, it has previously been demonstrated that reorganizing our inpatient care to be multidisciplinary has resulted in a reduction in below-the-knee amputations among patients with diabetic foot infections (19). Further supporting the need for a team of specialists treating ulcer patients, in this study ulcer patients had a 32-fold increased mortality risk from diabetes, and significantly increased risks were also detected for digestive diseases and ischaemic heart diseases. Ulcer patients moreover faced a 22-fold increased short-term risk of mortality from sepsis and mortality from pneumonia was also increased. The risk of short-term sepsis mortality in this study seemed even higher than earlier reports on all-cause mortality in chronic ulcer patients suggest (6, 8, 13, 15), which is essential to consider when treating patients with chronic ulcers.

### Strengths and limitations

The strengths of this study were a large and consecutively gathered cohort of chronic ulcer patients and their reference groups. Additionally, the study reported on patients with different aetiologies of chronic ulcers, including patients with atypical ulcers with very little previous mortality evidence, and the information on causes of death was from a reliable source. A further notable strength was the extensive duration of the study period spanning 4 decades, which made it possible to examine the changes in short-term mortality over time. Considering the limitations in our research, one of them was that because the data came from a hospital primarily focused on treating hard-to-heal ulcers, it may not fully reflect the entirety of the chronic ulcer patient population. Also, as a retrospective study, precise relationships between certain events cannot always be established, which needs to be considered when evaluating the results. Further, due to the available data we were unable to account for all confounding factors, such as presence of long-term illnesses and medications and also ulcer episodes before the study period, which might have influenced the results. Additionally, as in small subgroup analysis the confidence intervals were wide, future studies with larger cohorts are needed to confirm these findings.

### Conclusion

This study showed that patients with hard-to-heal chronic ulcers face a notably elevated short-term mortality risk, which is even higher than the previously reported long-term mortality. Those at the highest risk are patients with vasculitic, arterial, and PG ulcers, which emphasizes the necessity of prompt diagnosis and efficient treatment and follow-up of these patients in order to prevent adverse outcomes. Further, as mortality in this study was particularly associated with diabetes, and as the immediate risk of mortality from infection was increased, our results lend further support to the need for a multidisciplinary team of experts in the treatment of patients with chronic ulcers.

## Supplementary Material


