# Supplementary material for: Increases in One-year Mortality Risk Among Chronic Skin Ulcer Patients During the Period 1980–2020
Source: Acta Derm Venereol. 2026 Feb 10;106:43626. doi: 10.2340/actadv.v106.43626 (PMC12902911; doi:10.2340/actadv.v106.43626)
Supplement: Supplementary file 1 [file ActaDV-106-43626-s1.pdf]

Supplementary material has been published as submitted. It has not been copyedited, or typeset by Acta Dermato-Venereologica

Table SI. Hazard ratios (HR), 95% confidence intervals (CI) and statistical significances for one-year mortality in all ulcer patients and separately in females and males with different ulcer aetiologies during the period 1980-2020

	All	Female	Male
	HR (95% CI)	HR (95% CI)	HR (95% CI)
All Ulcers	3.8 (3.4-4.2)*	3.9 (3.4-4.4)*	3.6 (3.0-4.3)*
Venous Ulcers	2.0 (1.7-2.4)*	2.1 (1.7-2.7)*	1.9 (1.4-2.5)*
Arterial ulcers	7.0 (5.6-8.7)*	8.7 (6.2-12.1)*	5.8 (4.3-7.8)*
Mixed ulcers	3.7 (2.2-6.4)*	6.2 (2.9-13.3)*	2.1 (0.7-4.7)
Vasculitic ulcers	8.5 (5.7-12.6)*	8.5 (5.3-13.6)*	8.7 (4.0-18.6)*
PG ulcers	6.6 (3.0-14.7)*	11.5 (3.2-41.3)*	4.2 (1.4-12.0)*

*$p < 0.001$

HR: hazard ratios; CI: confidence intervals; PG: pyoderma gangrenosum.

Table SII. Hazard ratios (HR), 95% confidence intervals (CI) and statistical significances for one-year mortality in different ulcer aetiologies during the four study decades.

	1980-1989<br>HR (95% CI)	1990-1999<br>HR (95% CI)	2000-2009<br>HR (95% CI)	2010-1019<br>HR (95% CI)
All Ulcers	2.3 (1.8-3.1)*	3.0 (2.3-3.8)*	3.9 (3.2-4.9)*	4.9 (4.1-5.8)*
Venous ulcers	1.9 (1.4-2.6)*	1.5 (1.0-2.4)	2.2 (1.6-3.1)*	2.4 (1.7-3.4)*
Arterial ulcers	*NA*	3.7 (2.1-6.5)*	7.9 (5.1-12.2)*	7.6 (5.6-10.2)*
Mixed ulcers	*NA*	1.5 (0.1-16.7)	3.7 (1.5-9.0)*	4.1 (2.1-8.3)*
Vasculitic ulcers	1.5 (0.1-16.1)	8.8 (2.3-33.3)*	12.1 (4.5-32.5)*	8.3 (5.2-13.4)*
PG ulcers	*NA*	2.1 (0.4-12.4)	13.0 (2.8-61.3)*	7.0 (2.1-22.6)*

*$p < 0.01$

HR: hazard ratios; CI: confidence intervals; PG: pyoderma gangrenosum; *NA*: not applicable.
